# Experimental data of co-crystals of Etravirine and L-tartaric acid

**DOI:** 10.1016/j.dib.2017.11.019

**Published:** 2017-11-07

**Authors:** Mikal Rekdal, Aravind pai, Muddukrishna BS

**Affiliations:** a Department of Chemical Engineering, Norwegian University of Science and Technology, 7491 Trondheim, Norway; b Department of Pharmaceutical Quality Assurance, Manipal College of Pharmaceutical Sciences, Manipal University, Manipal, Karnataka 576104, India; c Department of Pharmaceutical Chemistry, Manipal College of Pharmaceutical Sciences, Manipal University, Manipal, Karnataka 576104, India

**Keywords:** Etravirine, Co-crystals, HPLC, FTIR instrument, DSC instrument

## Abstract

Etravirine is a drug used alongside other medication in the treatment of HIV and is a non-nucleoside reverse transcriptase inhibitor. It is a BCS class IV drug, having low solubility and high permeability (Drugbank, https://www.drugbank.ca/drugs/DB06414) [1]. As a result, large doses of the drug are required for treatment. Two pills have to be taken twice a day, making it a “pill burden” (Intelence, http://www.intelence.com/hcp/dosing/administration-options) [2]. Therefore, attempts of co-crystallizing Etravirine are attractive as the solubility of the drug tends to increase in this solid form (Schultheiss and Newman, 2009) [3].

In this study Etravirine co-crystals were synthesized in the molar ratios 1:1, 1:2 and 2:1 with L-tartaric acid as the co-former. Both slow evaporation and physical mixture was performed to mix the components. DSC values of final products are presented as well as FTIR spectra to observe the altered intermolecular interactions. A chemical stability test was performed after seven days using area under curve data from an HPLC instrument.

**Specifications Table**

**Table:** 

Subject area	*Pharmacy*
More specific subject area	*Pharmaceutical co-crystals*
Type of data	*Table and figure*
How data was acquired	*Fourier transform infrared (FTIR, Shimadzu FTIR-8300), differential scanning calorimeter (DSC, Shimadzu DSC-60) and high performance liquid chromatography (HPLC, Shimadzu LC-10 series) was used to analyze the product.*
Data format	*Analyzed, processed*
Experimental factors	– *Prepared co-crystals were stored in ambient conditions prior to analysis* – *Saturated solution was diluted by a factor of ten for solubility analysis within appropriate range*
Experimental features	*Preparation of co-crystals of Etravirine and L-tartaric acid in molar ratios 1:1, 1:2 and 2:1 with slow evaporation method. Solid state characterization of products using DSC and FTIR in addition to chemical stability analysis.*
Data source location	*Manipal, Karnataka, India*
Data accessibility	*Data are available in article*

**Value of the Data**

• Mixture of L-tartaric acid and Etravirine shows different IR spectra compared to pure drug.
• Further solubility studies of the co-crystals could investigate possible improved drug performance.
• Chemical stability proved for molar ratio 1:1 and 1:2 of Etravirine and L-tartaric acid.

## 1 Data

**Fig. 1:**
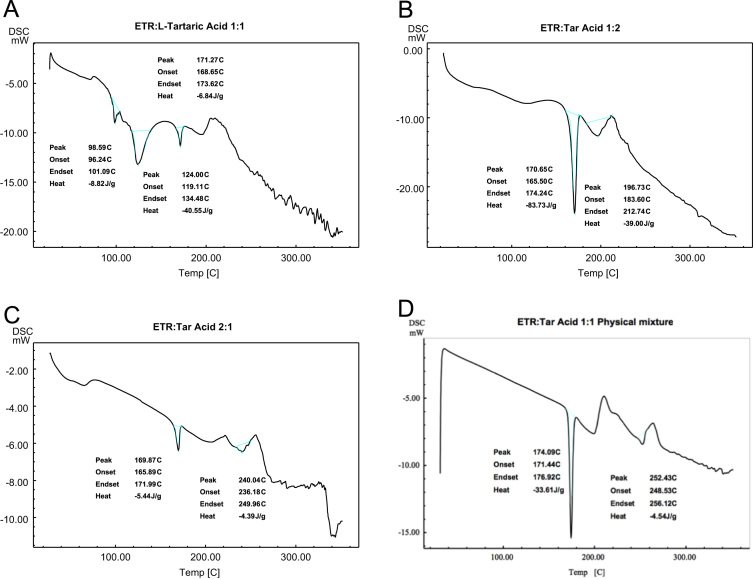
Thermograms from DSC analysis of products of Etravirine: L-tartaric acid (A) 1:1 by slow evaporation method, (B) 1:2 by slow evaporation method, (C) 2:1 by slow evaporation and (D) 1:1 by physical mixture.

**Fig. 2:**
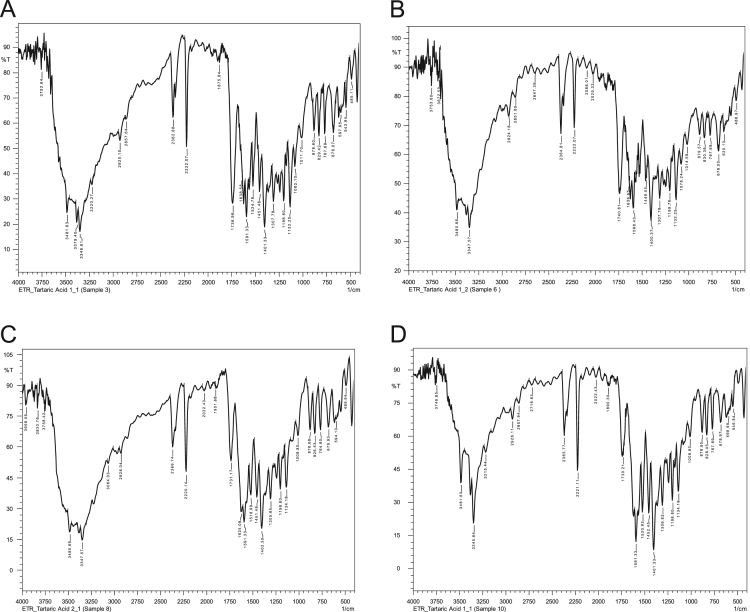
FTIR spectra of samples containing Etravirine: L-tartaric acid in molar ratios (A) 1:1 by slow evaporation method, (B) 1:2 by slow evaporation method, (C) 2:1 by slow evaporation* and (D) 1:1 by physical mixture. *Only one of the three batches of molar ratio 2:1 showed a broadened peak around 3500–3300 cm^−1^.

**Fig. 3:**
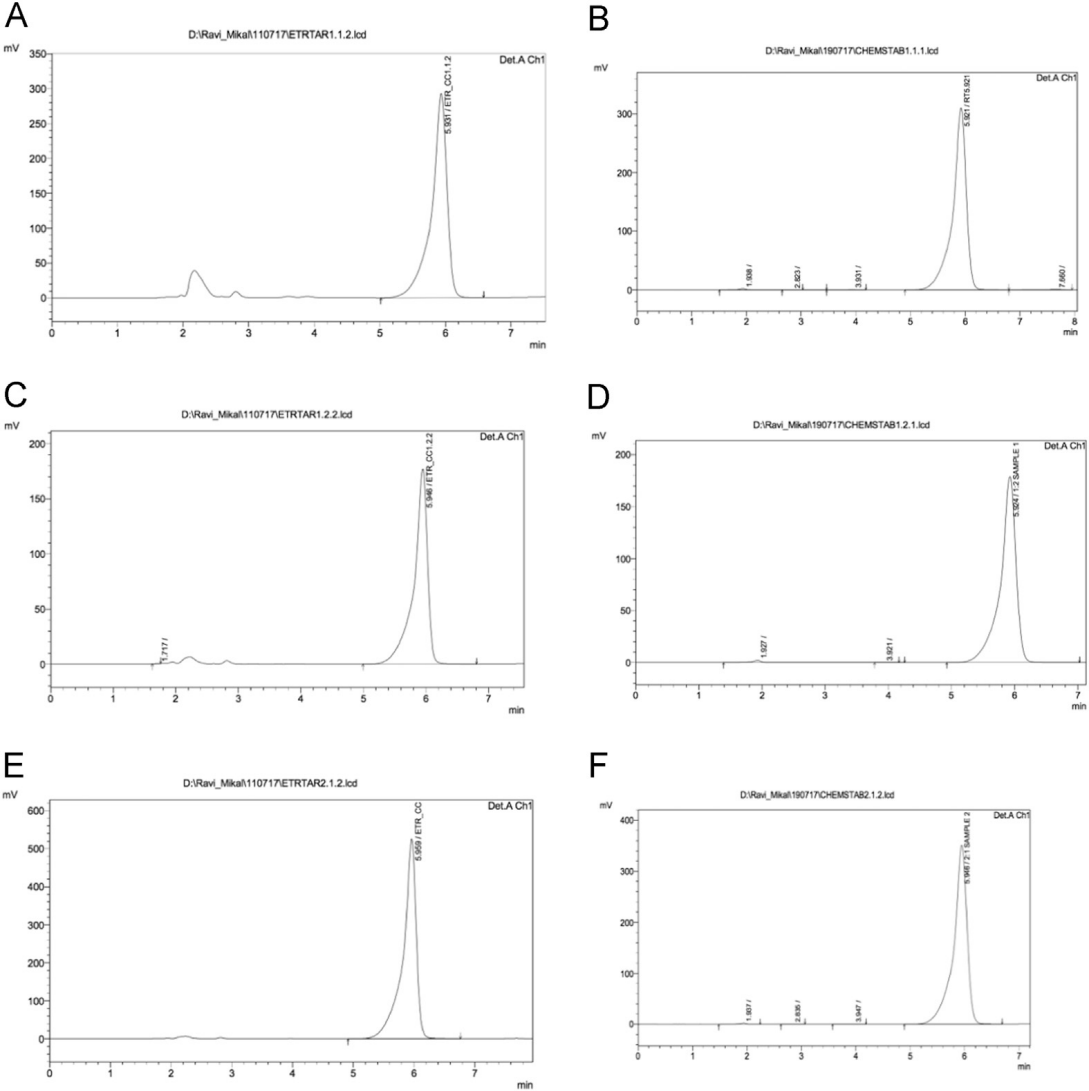
Chromatograms obtained from chemical stability analysis. A) 1:1, 0 days, B) 1:1, 7 days C) 1:2, 0 days D) 1:2, 7 days E) 2:1, 0 days F) 2:1, 7 days.

Data in this article shows the characteristics of products prepared from different molar ratios of Etravirine and L-tartaric acid. Table 3 shows the melting points of pure reactants and of the product samples and Fig. 1 displays the complementary thermograms. Fig. 2 displays the FITR spectra of the samples. All three sample batches prepared by slow evaporation method show a broadening of the primary amine peak (3300–3500 cm^-1^) whereas the physical mixture does not. Chemical stability data is shown in Fig. 3 and Table 4 where the retention peak for Etravirine is the only area of significant size for both 0 and 7 days.

## 2 Experimental design, materials, and methods

### 2.1 Materials

Etravirine was received from Apotex Research PVT LTD, Bangalore. L-tartaric acid was purchased from Sigma-Aldrich, Mumbai. HPLC grade acetonitrile was obtained and analytical grade methanol was obtained from FINAR Limited, Ahmedabad and acetone from Merck Life Sciences Private Limited, Mumbai. A Milli-Q purification system (Siemens AG, Germany) was used in the laboratory to obtain HPLC grade water.

L-tartaric acid was selected as co-former after promising results in increased solubility with DL-tartaric acid as co-former from a research study performed at Manipal College of Pharmaceutical Sciences during the fall of 2016 [4]. Etravirine has 2 hydrogen bond donor sites and 7 hydrogen bond acceptor sites resulting in a high probability of co-crystal formation with co-formers.

### 2.2 Co-crystal preparation

Co-crystals were prepared in molar ratios 1:1, 1:2 and 2:1 of Etravirine and L-tartaric acid respectively through slow evaporation method. Desired amount of co-crystal component was weighed and saturated solutions of the components were prepared separately by adding approximately 1 mL solvent. The solvent used was acetone: methanol (50:50% v/v). The saturated solutions were mixed in a common vial and vortexed for 10 minutes. After, the solution was spread evenly on a petri dish and covered with aluminum foil with holes. The solution evaporated at room temperature until the sample was completely dry. The petri dish was scraped and the co-crystals were collected and stored for further analysis. 500 mg of co-crystals were produced in triplicate batches (*n*=3) for each molar ratio as described in Table 1.

**Table 1:** Mass needed for 500 mg co-crystals in different ratios and number(name) of samples.

**Etravirine:L-tartaric acid ratio**	**Etravirine [mg]**	**L-tartaric acid [mg]**	**No. of samples prepared**
1:1	371.7	128.3	3
1:2	296.0	204.0	3
2:1	426.5	73.5	3
1:1 physical mix.	371.7	128.3	1

A single physical mixture sample of molar ratio 1:1 was also prepared in a plastic vial by mixing with a micro spatula for two minutes and then shaking the vial manually for five minutes.

### 2.3 Analysis

1. DSC
  Shimadzu DSC-60 was used for obtaining DSC values. A single DSC sample consisting of all three batches was analyzed for each molar ratio. A sample of the physical mixture was also prepared, making 4 samples in total. 5 mg of each sample was placed in an aluminum bottom and crimped with an aluminum top. The test ran in the temperature range 30–350 °C with a temperature increase of 10 °C/min.

2. FTIR
  A Shimadzu FTIR-8300 was used to acquire the FTIR spectra of the co-crystals. One FTIR sample per batch was prepared in addition to the physical mixture making 10 samples in total. The samples were dispersed in KBr which was then grinded to a disk by applying pressure. The measured range was 4000–500 cm^−1^ with 25 scans.

3. HPLC
  The co-crystal purity was analyzed with a Shimadzu LC-10 series chromatographic system. The system contained a controller unit (SCL-10A VP), a degasser unit (DGU-20A5), a quaternary gradient pump (LC-20AD), a refrigerated auto sampler (SIL-20AC HT) and a PDA detector (SPD-20A). The buffer solution was filtered using a vacuum-filtration apparatus (Alltech Associates) with a 0.45 μm filter (Pall Life Sciences). The mobile phase was degassed by sonication in Equitron ultrasonic bath. For the stationary phase a Hypersil BDS C_18_ (150×4.6 mm×5 μm) column was used and the mobile phase was a mixture of acetonitrile: phosphate buffer (60:40% v/v). The flow rate was 1 mL/min and detection wavelength at 304 nm at 30 °C.

### 2.4 HPLC sample preparation

The equivalent of 10 mg Etravirine in each co-crystal form was weighed out from a mixture of product batches with the same molar ratios. A stock solution was created by adding amount from Table 2 in 10 mL acetonitrile: methanol (50:50% v/v). 0.5 mL was pipetted from stock and diluted to 10 mL with the same solvent. 0.3 mL of diluted solution was further diluted with 1.2 mL acetonitrile: phosphate buffer (60:40% v/v). The product analysis was performed in triplicate (*n*=3) for each molar ratio.

**Table 2:** Mass of co-crystals needed for the equivalent amount of 10 mg Etravirine.

**ETR:TAR composition**	**Mass co-crystals weighed [mg]**
1:1	13.5
1:2	16.9
2:1	11.7

**Table 3:** Literature values for melting points of components and DSC values for the co-crystal preparations. ETR is Etravirine, TAR is L-tartaric acid and CC stands for co-crystal.

**Sample**	**Melting endotherms [°C]**
Etravirine	260.36 [5]
L-tartaric acid	171–174 [6]
1:1 ETR:TAR CC	95.59 & 124.00 & 171.27
1:2 ETR:TAR CC	170.65 & 196.73
2:1 ETR:TAR CC	169.87 & 240.04
1:1 ETR:TAR physical mix	174.09 & 252.43

**Table 4:** HPLC data for average area under curve for the Etravirine peak initially and after 7 days, as well as percentage assay.

**Co-crystal sample**	**Average area under curve, 0 days (*n*=3)**	**Average area under curve, 7 days (*n*=2)**	**% Assay**
1:1	4827348	5333824	110,5
1:2	2663206	3063559	115,0
2:1	7999479	6047109	75,6

### 2.5 Stability studies

After 7 days, an additional HPLC test was run as described above on the co-crystals to examine their chemical stability and the possible appearance of degradation products. Samples were prepared in duplicates (*n*=2) for each molar ratio.
